# Foot-Mounted Pedestrian Navigation Method by Comparing ADR and Modified ZUPT Based on MEMS IMU Array

**DOI:** 10.3390/s20133787

**Published:** 2020-07-06

**Authors:** Li Xing, Xiaowei Tu, Zhi Chen

**Affiliations:** School of Mechatronic Engineering and Automation, Shanghai University, Shanghai 20444, China; xinglishu@shu.edu.cn (L.X.); tuxiaowei@shu.edu.cn (X.T.)

**Keywords:** pedestrian navigation, MEMS IMU array, adaptive dead reckoning (ADR), zero velocity update (ZUPT)

## Abstract

Using an MEMS Inertial Measurement Unit (MEMS IMU) array mounted on foot is a feasible approach to improve the pedestrian tracking accuracy for the pedestrian navigation system (PNS). Based on the in-house developed IMU array, the paper proposes a new integrated framework that combines adaptive deck reckoning (ADR) with the modified zero velocity update (ZUPT). In the proposed ADR algorithm, the IMUs with large drift errors on the array are selected and removed according to the step length and the track angle computed by each IMU on the array. Then, by using the step length and the track angle of each step computed by remaining IMUs, the foot position extracted from the traditional ZUPT model is estimated on the basis of least squares (LS) so as to improve the traveled distance calculation accuracy. Compared with the traditional IMU array fusion method based on a maximum likelihood estimator (MLE) when it is used in the PNS, which is approximately taking the mean value of array readings, the proposed method is equivalent to adaptively fusing the array readings and thus improves the pedestrian tracking accuracy. To compare the proposed method with MLE, two different types of walking tracks are designed. The 161 m straight line experiments show that the end position by ADR/modified ZUPT method is much closer to the one of the reference trajectory compared with the MLE in repeated walks, and the closed-loop tracks about 300 m show that the positioning error with respect to the total traveled distance is less than 0.6% (1σ), which is higher than 1% (1σ) of MLE.

## 1. Introduction

Pedestrian navigation systems (PNS) have drawn much attention in recent years because they can be of significant use in applications for both civilian and military [1,2], especially in indoor environments where GPS-like satellite external positioning is degraded or unavailable [3,4,5]. Pedestrian navigation technologies can be divided into two sorts: the infrastructure-based positioning technology and the infrastructure-free navigation solutions [6,7]. When fire, earthquake, or other emergency occurs, these communication infrastructures (wireless fidelity (WIFI) [8], bluetooth [9], ultra-wide-band (UWB) [10,11], ZigBee [12]) would be damaged, thus resulting in many restrictions. The infrastructure-free navigation solution mainly applies some self-contained on-body sensors (usually including three accelerometers, three gyroscopes, and three magnetometers) to locate the position without external facilities. However, when the infrastructure-free navigation solution applies magnetometers to estimate the yaw [13], there is an issue that the estimation would be easily affected by the magnetic disturbance. Thus, the MEMS inertial measurement unit (IMU) only including accelerometers and gyroscopes with the independence attracts the attention of many researchers [14,15,16,17,18].

MEMS IMU is often attached on shoes, and the so-called foot-mounted navigation system [19,20] becomes the main option for PNS. However, MEMS IMU has a low measurement precision and a drift problem in which errors accumulate over time [21]. Thus, the zero velocity update (ZUPT) algorithm has been proposed and widely used to mitigate the accumulated error [22]. The elegance of the framework lies in the fact that the foot swings to stance phase periodically during most types of human locomotion, such as walking, running, and ascending or descending stairs [23,24]. Once a stance phase is detected, the error of velocity output and zero velocity can be employed as the pseudo measurement so as to correct accumulated errors.

Nevertheless, ZUPT using only one IMU mounted on only one foot cannot ensure the measured distance at an acceptable level of accuracy [25,26,27]. In order to reduce the diverging position error, some aided correction sensors or methods are applied. The foot-mounted IMU and radio frequency identification (RFID) measurements tightly coupling method proposed in [28] places several active RFID tags at known locations in a building and corrects the diverging position error by the received signal strengths (RSSs). However, such an aided method faces the same problem as the infrastructure-based positioning technology, that is, RFID tags would be damaged in a few complex and hostile environments and be unable to be used. Increasing the number of inertial sensors has been proposed as an efficient idea to achieve better positioning performance. These solutions include a dual-foot PNS, dual-IMU PNS on one foot and pedestrian tracking using an IMU array. The principle of the dual-foot PNS or the dual-IMU PNS is based on a physical constraint on the position and the attitude information in the course of a pedestrian movement. For example, ref. [29] aims to reduce the position error by using RDUPT (relative distance update) or RVUPT (relative vector update) to deal with the data from two inertial systems located on each foot, and [30] adopts a projecting method to solve the problem with an upper bound of maximum spatial separation, which adds an inequality constraint model to the traditional ZUPT. In addition to adding the inequality constraint model to the traditional ZUPT, the measured distance between feet or dual IMUs on one foot by ultrasonic wave [31] or radio frequency (RF) signal is added to the ZUPT measurement model so as to reduce the position error. However, for these systems, when two IMUs on dual feet or one foot have the same error drift trend, the relative distance constraint could be still satisfied and thus could not effectively correct the drift error of the position. An IMU array that combines multiple IMUs on a single PCB has recently been demonstrated; their measurements can be combined to mitigate independent stochastic errors of IMU. Additionally, using an array of ultra-low-cost IMUs is a feasible approach to improve performance for foot-mounted inertial navigation systems, which is demonstrated in [32,33,34].

In the method of pedestrian tracking using an IMU array, the key problem is how to fuse multiple IMUs to attain the potential performance gain. Ref. [34,35] presents a maximum likelihood estimator (MLE) for fusing the measurements in an inertial sensor array, and ref. [36] extends the maximum likelihood estimator by introducing a motion model and deriving a maximum a posteriori estimator for estimating angular velocity, angular acceleration, and specific force of an inertial sensor array. Ref. [37] applied the maximum likelihood estimator (MLE) of the IMU array on the pedestrian tracking tests, and the results show that such a method can give suboptimal performance on the pedestrian tracking and how to weight the IMU measurements to attain better pedestrian tracking performance is not clear.

The main study of this paper is to develop a foot-mounted IMU array fusion frame and improved ZUPT filtering model in order to attain better multi-IMU measurements fusion performance and then improve the pedestrian position estimation accuracy. In the proposed adaptive pedestrian dead reckoning method, the position error is separated from the traditional ZUPT filtering model, and the pedestrian position is estimated by an adaptive deck reckoning (ADR) algorithm. In the proposed ADR algorithm, firstly, IMUs with large drift errors are selected and removed according to the step length and the track angle computed by every IMU in the array during a ZUPT period. Then, on the basis of least squares (LS), step length and track angle of each step are estimated synthetically by using that computed by remaining IMU; therefore, every step position of the pedestrian is calculated through ADR algorithm. Lastly, the performance advantages of the proposed ADR/ZUPT integrated framework over the MLE method are verified by pedestrian positioning accuracy in different track tests.

The innovations in this paper are mainly reflected in the following two aspects:

(1) Aiming at the problem that the MLE method for fusing IMU array data has difficulty balancing reasonably the influence exerted by every IMU to the pedestrian navigation accuracy, a fusion scheme of IMU with large drift error elimination using the position calculated by each IMU is proposed in this paper.
(2) In order to further improve the estimation accuracy of the position in the traditional ZUPT filtering model, the position estimator is extracted from its filtering model using adaptive dead reckoning based on LS to carry out the independent estimation.

The remainder of this paper is organized as follows. Section 2 gives a detailed description of the ADR/ZUPT integrated framework based on the adaptively fusing foot-mounted IMU array. The experimental equipment and the results are shown in Section 3. Conclusions are presented in Section 4.

## 2. ADR/ZUPT Integrated Framework Based on Adaptively Fusing Foot-Mounted IMU Array

In this section, we give a brief description of the in-house developed MEMS IMU array in Section 2.1. Then, the ADR/ZUPT integrated algorithm structure is described in Section 2.2, where the differences between the MLE based on the traditional ZUPT and the proposed ADR/modified ZUPT are analyzed. Section 2.3 discusses the position estimation process by adaptively fusing foot-mounted IMU array in the ADR module in details, which is the core innovation of the proposed method.

### 2.1. In-House Developed MEMS IMU Array

**Figure 1:**
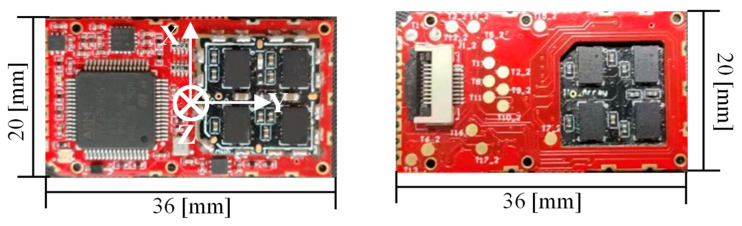
The in-house developed inertial measurement unit (IMU) array platform.

The in-house developed IMU array is shown in Figure 1. The array holds eight MEMS IMUs (four on the top side and four on the bottom side) and a STM32F405 microcontroller. The displayed photo is the actual size of the platform. Such a designed structure is lightweight and can be conveniently attached to the heel of the user’s boot. The IMUs on the array have been compensated for temperature sensitivities to bias and scale factor, nonorthogonal coupling, and misalignment errors. When the foot-mounted array is used for the pedestrian navigation, two coordinate frames should be introduced. The body coordinate frame (b-frame for short) is parallel to the sensor’s axes shown in Figure 1. The navigation coordinate frame (n-frame for short) is a local east-north-up reference frame. All the IMU data are measured in the b-frame, while all the inertial navigation system (INS) states are calculated in the n-frame.

### 2.2. ADR/ZUPT Integrated Algorithm Structure

**Figure 2:**
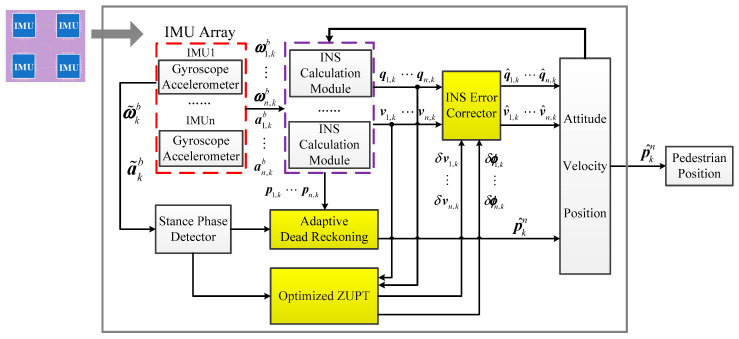
Block diagram of the adaptive deck reckoning (ADR)/modified zero velocity update (ZUPT) integrated algorithm.

The ADR/ZUPT integrated algorithm structure proposed in this paper has five modules: INS calculation module, stance-phase detector, adaptive dead reckoning module, optimized ZUPT filtering module, and INS error corrector, as shown in Figure 2. The INS calculation module and the stance-phase detector have been in the traditional ZUPT [23]. Compared to the traditional ZUPT algorithm, the adaptive dead reckoning module is added in the proposed integrated structure. ZUPT filtering model and INS error corrector are optimized. The functionalities of these three modules are described below.

(1) Adaptive dead reckoning module: As the traditional ZUPT filter model observes the position error weakly, the paper separates the position error from the filter model and fuses the IMU array redundant measurements to estimate the pedestrian position by applying the dead reckoning algorithm. In order to reasonably weight the IMU measurements to attain better position fusion performance, the paper proposes an adaptive dead reckoning (ADR) algorithm based on least squares (LS) to estimate the pedestrian position, which is detailed in the next section.
(2) Optimized ZUPT module: The traditional ZUPT module uses the Kalman filter as an indirect filter to estimate the errors of the raw INS states. As the position is estimated in the adaptive dead reckoning module separately, the optimized ZUPT process filter model only contains the velocity error ${\delta \nu }^{n}$ and the attitude error δϕ. An individual optimized ZUPT module is established for each IMU of the array. Since the sampling period Δt is very small, the continuous-time error model of *i*: th IMU can be discretized as
  (1) $$\begin{array}{c} {\delta x}_{i,k}=\Phi_{i,k}\delta x_{i,k-1}+\Gamma_{i,k}w_{i,k-1},\\ \Phi_{i,k}=I+F_{i,k}\Delta t,\\ \Gamma_{i,k}=G_{i,k}\Delta t, \end{array}$$
  where ${\delta x}_{i,k}={\left[{{\delta \nu }_{i,k}^{n}}^{T}{\delta \phi }_{i,k}^{n}{}^{T}\right]}^{T}$, I is a 6 × 6 identity matrix, $F_{i,k}=\left[\begin{array}{cc} 0_{3\times 3} & \left(C_{b}^{n}\cdot a_{i,k}^{b}\right)\times \\ 0_{3\times 3} & 0_{3\times 3} \end{array}\right]$ and $G_{i,k}=\left[\begin{array}{cc} C_{b}^{n} & 0_{3\times 3}\\ 0_{3\times 3} & -C_{b}^{n} \end{array}\right]$. $w_{i,k-1}$ is zero mean white noise with covariance matrix $Q_{i,k}=E\left[w_{i,k}w_{i,k}{}^{T}\right]$. Since the noise of accelerometers and gyroscopes in our designed system is respectively 0.01 m/s^2^ and 0.005 rad/s, $Q_{i,k}$ is a 6 × 6 diagonal matrix with diagonal elements as $\left[\begin{array}{cc} {\left(0.01\right)}_{1\times 3}^{2} & {\left(0.005\right)}_{1\times 3}^{2} \end{array}\;\right]$.
  The measurement model of *i*:th IMU can be expressed as
  (2) $$z_{i,k}=H\delta x_{i,k}+\eta_{i,k},$$
  where $z_{i,k}=v_{i,k}^{n}$ and the measurement matrix $H=\left[\begin{array}{cc} I_{3\times 3} & 0_{3\times 3} \end{array}\;\right]$. The measurement noise $\eta_{i,k}$ is zero mean white noise with covariance matrix $R_{i,k}=E\left[\eta_{i,k\;}\eta_{i,k}{}^{T}\right]$. $R_{i,k}$ is set to be a 3 × 3 diagonal matrix with elements as $\left[{\left(0.005\right)}_{1\times 3}^{2}\right]$ based on experimental results.
(3) INS error corrector: The error corrector utilizes the error estimates to refine the raw INS states and suppress the cumulative divergence error. Different from the INS error corrector in the traditional ZUPT, such a module only corrects attitude and velocity errors. The error corrector model of the *i*:th IMU is given by
  (3) $$\begin{array}{c} {\hat{C}}_{i,b_{k}}^{n}=\left[I_{3\times 3}-\Theta_{i,k}\right]C_{i,b_{k}}^{n},\\ {\hat{C}}_{i,b_{k}}^{n}\Rightarrow {\hat{q}}_{i,k},\\ {\hat{v}}_{i,k}^{n}=v_{i,k}^{n}-\delta v_{i,k}^{n}, \end{array}$$
  where ${\hat{C}}_{i,b_{k}}^{n}$ is the corrected attitude matrix, ${\hat{q}}_{i,k}$ is the corrected attitude quaternion, $\Theta_{i,k}$ is the skew-symmetric matrix of δϕ, and ${\hat{v}}_{i,k}^{n}$ is the corrected velocity.

### 2.3. Position Estimation by Adaptive Dead Reckoning Based on IMU Array

**Figure 3:**
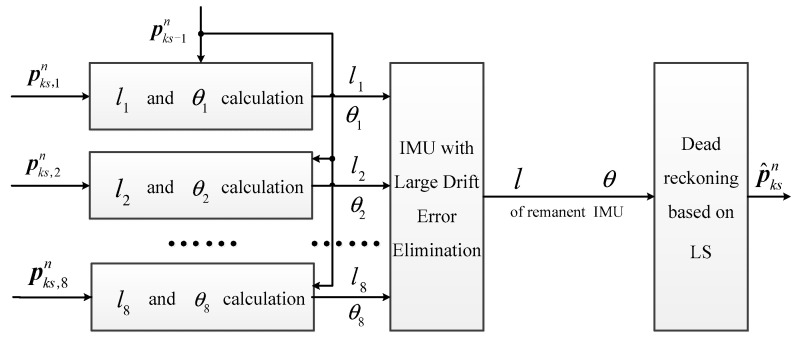
Position estimated method by adaptive dead reckoning.

In ref. [35], the IMU array is applied in the pedestrian navigation system with the MLE method, which is equivalent to fuse the gyro and the accelerometer output with equal weight, whose position accuracy is theoretically superior to that of a single IMU. However, in the repeated test of a real system, sometimes the pedestrian position accuracy of IMU array is lower than that of a single IMU, which is a result of the difference in drift errors of each IMU. Therefore, the fusion result is affected by IMUs with large drift errors, with the measurement accuracy of angular rate and acceleration after fusion being lower than that of the IMU with minimum drift errors. To further improve the pedestrian position estimation accuracy of MLE, this section proposes the position estimated method by adaptive dead reckoning, whose basic idea is shown in Figure 3.

**Figure 4:**
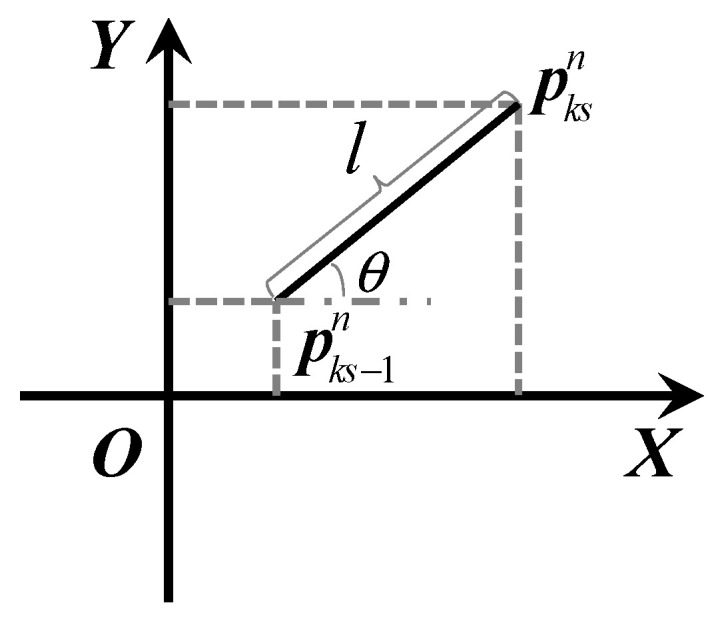
The principle of deck reckoning method.

In Figure 3, $p_{ks-1}^{n}=\left[p_{x,ks-1}^{n},p_{y,ks-1}^{n}\right]$ represents the horizontal position estimated in the (ks−1): th step, while $p_{ks,1}^{n}\ldots p_{ks,8}^{n}$ represent the position independently calculated by every IMU in the array module in the ks: th step. $l_{1}\ldots l_{8}$ are the step lengths calculated by each IMU in the ks:th step, and $\theta_{1}\ldots \theta_{8}$ are track angles. The two variables are calculated according to the principle of deck reckoning method shown in Figure 4.

By using the step lengths and the track angles of each IMU calculated in Equation (4), comparison between drift errors can be made, thus eliminating the IMU with large drift errors. Then, through the optimization fusion of l and θ, the position estimation of every step can be realized. The module “IMU with Large Drift Error Elimination” in Figure 3 is discussed in detail in Section 2.3.1. On this basis, Section 2.3.1 proposes the dead reckoning method based on LS.

(4) $$\begin{array}{c} p_{x,ks}^{n}=p_{x,ks-1}^{n}+l\cos\theta ,\\ p_{y,ks}^{n}=p_{y,ks-1}^{n}+l\sin\theta \end{array}$$

#### 2.3.1. Elimination of IMU with Large Drift Errors

**Figure 5:**
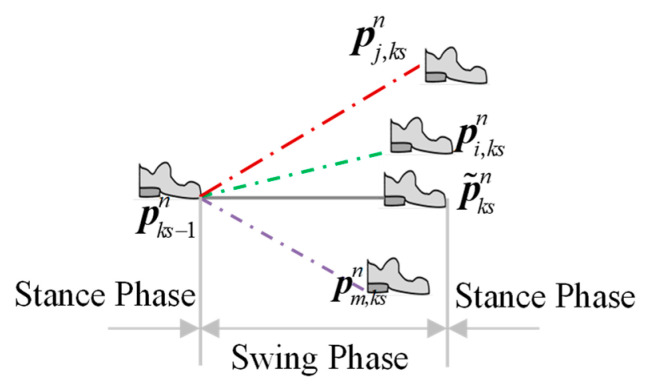
Pedestrian positions calculated by IMU with different drift errors during a step.

Although it is difficult to make comparison between drift errors from the gyro and the accelerometer outputs of IMU, differences appear in the position’s solution after integration. After the zero speed detection, the pedestrian walking process can be divided into several stance phases and swing phases. If every IMU in the former stance phase is in the same position, then, after the strapdown inertial navigation solution of swing phase, due to the difference between drift errors, the IMU is in different positions in the next stance phase, which is shown in Figure 5, where ${\tilde{p}}_{ks}^{n}$ is the ideal position.

When the maximum drift error exists in the ith gyroscope, there is a maximum deviation between the track angle $\theta_{i}$ and the ideal one θ, which is due to the certain relationship between the track angle and the yaw, and the yaw error is mainly effected by gyro drift errors. Although the ideal track angle is inaccessible in the solution process, the IMU label with maximum gyro drift errors can be distinguished through

(5) $$\Delta \theta_{\mathrm{sum},i}=\sum_{k=1,k\neq i}^{n}\left|\theta_{i}-\theta_{k}\right|$$

When the maximum drift error exists in the ith accelerometer, since the step length approximates to the double integral of acceleration, there is a maximum deviation between the calculated step length $l_{i}$ and the ideal one l, that is to say, the sum $\Delta l_{\mathrm{sum},i}$ of the step-length difference calculated by the ith IMU and other IMUs is the largest. The formula of $\Delta l_{\mathrm{sum},i}$ is shown in

(6) $$\Delta l_{\mathrm{sum},i}=\sum_{k=1,k\neq i}^{n}\left|l_{i}-l_{k}\right|.$$

**Figure 6:**
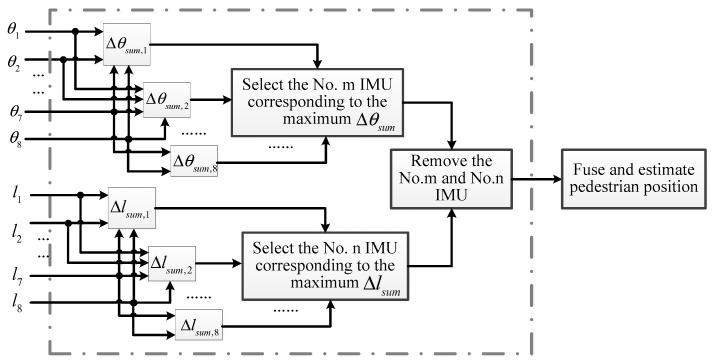
Pedestrian positions calculated by IMU with different drift errors during a step.

Therefore, the IMU with the largest drift error can be eliminated according to the calculation of $\Delta \theta_{\mathrm{sum}}$ and $\Delta l_{\mathrm{sum}}$. The selecting and removing process is depicted in Figure 6.

In the walking process, the adaptive removal of IMU with large drift errors can eliminate its influence on other IMUs when carrying out position fusion, thus resulting in the improvement of IMU array accuracy when estimating pedestrian position. On this basis, the method of fusing the step length and the track angle calculated by the IMU array in each step to recursively get the pedestrian position is analyzed in the next section.

#### 2.3.2. Dead Reckoning Based on Least Squares

Based on the elimination of IMU with large drift errors, this section carries out the fusion of step lengths and the track angles in each step by LS. Therefore, the fusion position with the minimum error variance can be estimated.

According to Equation (4), the horizontal position $p_{ks,i}^{n}$ calculated in the ks:th step by the ith IMU can be written into Equations (7) and (8),

(7) $$p_{ks,ix}^{n}=p_{ks-1,ix}^{n}+\left(l+\Delta l_{i}\right)\cos\left(\theta +\Delta \theta_{i}\right)$$

(8) $$p_{ks,iy}^{n}=p_{ks-1,iy}^{n}+\left(l+\Delta l_{i}\right)\sin\left(\theta +\Delta \theta_{i}\right)$$

where l is the ideal step length and θ is the ideal track angle. $\Delta l_{i}$ is the step length error calculated by the i th IMU and $\Delta \theta_{i}$ is the track angle error. Through ignoring the error of Taylor expansion, we can obtain the formula shown in Equations (9) and (10), where ε is the second order small quantity.

(9) $$p_{ks,ix}^{n}=p_{ks-1,ix}^{n}+l\cos\theta -\Delta \theta_{i}l\sin\theta +\Delta l_{i}\cos\theta +\varepsilon$$

(10) $$p_{ks,iy}^{n}=p_{ks-1,iy}^{n}+l\sin\theta -\Delta \theta_{i}l\cos\theta +\Delta l_{i}\sin\theta +\varepsilon$$

The position of every multi inertial measurement unit (MIMU) can be written into the formula shown in Equations (9) and (10). Therefore, after making a difference between each two, we can obtain Equation (11), where n is the number of remaining MIMU after the large drift error elimination, and the subscript (n−1)n denotes the parameter of the (n−1)th MIMU minus that of the nth MIMU. Similarly, a series of equations about $p_{ks,\left(i-1\right)iy}^{n}$ can be derived based on Equation (10).

(11) $$\begin{array}{c} p_{ks,12x}^{n}=\Delta l_{12}\cos\theta -\Delta \theta_{12}l\sin\theta +\varepsilon \\ p_{ks,13x}^{n}=\Delta l_{13}\cos\theta -\Delta \theta_{13}l\sin\theta +\varepsilon \\ \vdots \\ p_{ks,\left(n-1\right)nx}^{n}=\Delta l_{\left(n-1\right)n}\cos\theta -\Delta \theta_{\left(n-1\right)n}l\sin\theta +\varepsilon \end{array}$$

Then, they can be simplified into

(12) $$\begin{array}{c} \Delta P_{x}=A_{x}X_{x}+\Delta \varepsilon ,\\ \Delta P_{y}=A_{y}X_{y}+\Delta \varepsilon \end{array}$$

where $X_{x}={\left[\begin{array}{cc} \cos\theta & l\sin\theta \end{array}\right]}^{T}$. The coefficient matrix $A_{x}$ and $A_{y}$ can be calculated by step length and track angle calculated in each IMU, as is shown in

(13) $$\begin{array}{c} \Delta l_{k}-\Delta l_{j}=\left(l+\Delta l_{k}\right)-\left(l+\Delta l_{j}\right)=l_{k}-l_{j},\\ \Delta \theta_{k}-\Delta \theta_{j}=\left(\theta +\Delta \theta_{k}\right)-\left(\theta +\Delta \theta_{j}\right)=\theta_{k}-\theta_{j} \end{array}$$

$X_{x}$ or $X_{y}$ can be solved by least square method from (13).

Substituting the lsinθ calculated in $X_{x}$ and the lcosθ in $X_{y}$ into Equation (4), the pedestrian position can be calculated recursively.

To sum up, compared with the traditional method, the proposed pedestrian navigation method based on foot-mounted IMU array adaptively selects and removes the influence exerted by the IMU with large drift errors on position estimation. It estimates the pedestrian position independently based on LS, solving the problem of low accuracy caused by the weak observability of position estimation in the traditional ZUPT model. The performance advantages of the proposed method are verified and analyzed through multiple walking track tests in Section 3.

## 3. Experiments and Analysis

In order to analyze the performance of the proposed foot-mounted pedestrian navigation method by comparing ADR and modified ZUPT, we designed two types of walking track, which are as follows: (1) straight line with a length of approximately 160 m; (2) closed-loop track with a total length of approximately 300 m.

**Figure 7:**
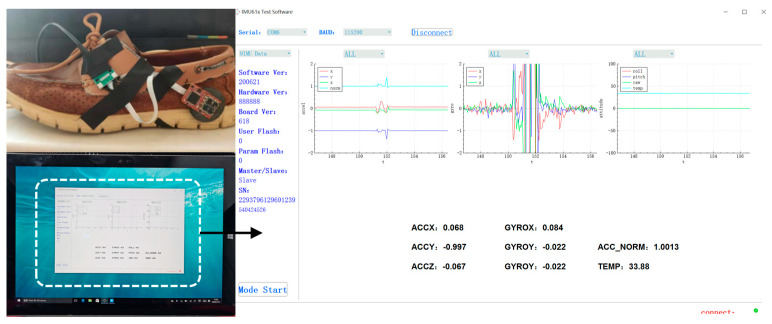
Walking experiments devices.

The experiment devices include the in-house developed MEMS IMU array shown in Figure 1 and the designed data acquisition software on the laptop. The IMU array module is installed on the heel and connected with the laptop through a USB data cable shown in Figure 7. The data are collected and stored with 400 Hz, which includes gyroscope and accelerometer measurements of each IMU on the array and the fusion IMU data by the MLE method. Then, the data are analyzed offline and used to estimate the pedestrian position by the traditional ZUPT and the proposed ADR/ZUPT integrated framework, respectively. The data acquisition software can show the triaxial accelerometer and the gyroscope real-time output of the fusion IMU by the MLE and whether IMUs are running normally, as shown in Figure 7. Seen from the IMU output of the software, we can also judge whether the pedestrian is walking or standing still.

To be specific, there is a reference trajectory in Track 1, and the method performance is evaluated based on the walking position error of the estimation end point and the reference end point. As for Track 2, the performance evaluation is based on the distance between the end point and the start point in the track. In the designed walking track, the pedestrian position is estimated respectively by adopting the maximum likelihood estimator (MLE) method and the proposed ADR/ZUPT integrated framework, then the performance of the proposed method in pedestrian location is compared and analyzed.

### 3.1. Straight Line Experiments

**Figure 8:**
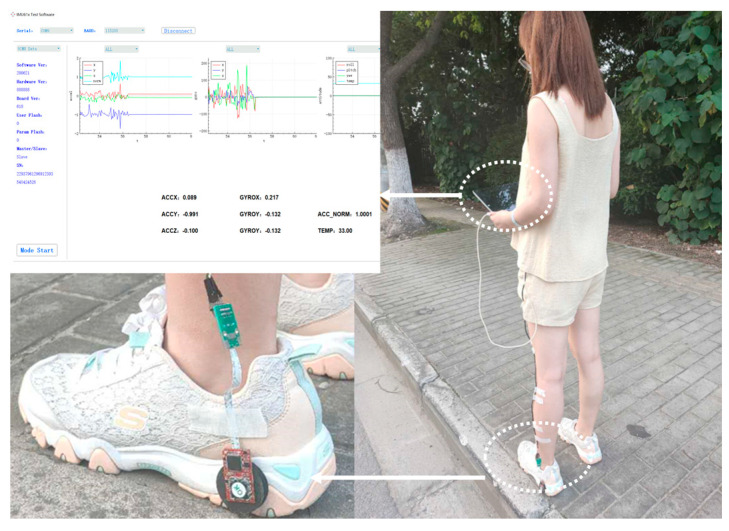
Straight line experiments scene based on foot-mounted IMU array.

**Figure 9:**
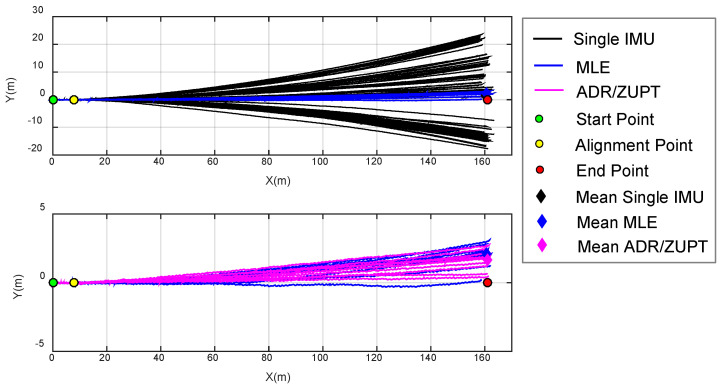
Estimated trajectories of different methods in 10 straight line walking experiments.

In the straight line experiments, we chose a section of curbstones at the roadside as the reference track. The total length ias measured by the number of curbstones, whose length was approximately 1 m, and the total length was 161 m. The walking scene is shown in Figure 8. The initial orientation was set by first letting experimenter walk about 9 m between the start point and the ninth curbstone (green and yellow circles in Figure 9), which were marked before testing. The experimenter remained standing in the two places for a period so as to identify the location of the ninth curbstone. The estimated translation was then used as a base line defining zero heading direction. Thus, all the estimated trajectories were aligned with the initial orientation of the reference trajectory. In the tests, the same walk and orientation was repeated 10 times. We respectively used the single IMU on the array and the fusion IMU by MLE and ADR/ZUPT to estimate the walking tracks, and the estimation results are shown in Figure 9.

Based on the results of Figure 9, the statistical errors of the end position between estimated and reference tracks are calculated in Table 1. Table 1 compares the end position estimation errors with different methods in each walk test and respectively gives the mean errors in the 10 repeated walks.

**Figure 10:**
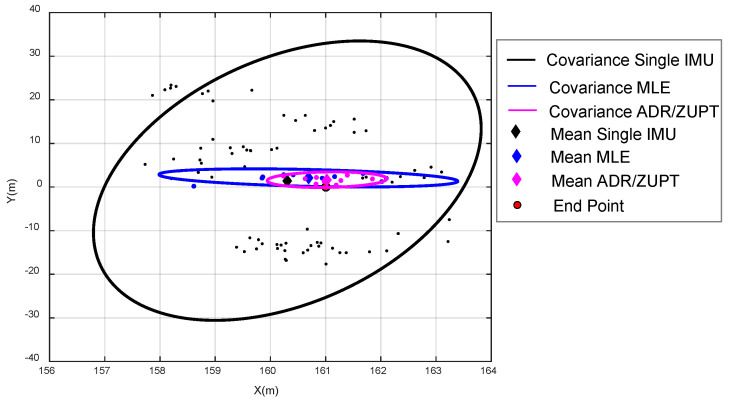
The covariance and mean of the estimated end position by different methods.

We also analyzed the covariance of the end position by the single IMU, MLE, and ADR/ZUPT. Their distributions are shown in Figure 10. The 95% confidence ellipse of different methods is respectively plotted.

Seen from the estimated tracks in different colors in Figure 9 and Figure 10 and Table 1, we can come to the conclusions as follows:

(a) The 10 walking tracks symmetrically diverge based on the reference trajectory, which demonstrates that deterministic errors of single IMU on the array are effectively calibrated and compensated.
(b) Although the bias of each IMU is deducted online after powering on, online drift errors still exist in the process, and the drift errors of each IMU differ from each other, which results in the difference in the estimated walking track. The maximum drift error is about 25 m in the vertical direction of walk trajectories.
(c) Due to the randomness of the drift errors of each IMU at different times, their calculated tracks sometimes get close and sometimes depart from the ideal track. Therefore, it is required to make adaptive comparison of drift errors according to the calculated position of each IMU at different times, which verifies the rationality of the adaptive elimination method of IMU with large drift errors in the ADR/ZUPT integrated framework.
(d) There is clearly a gain by combining multiple IMUs, whether based on MLE or ADR/ZUPT. In repeated experiments, the maximum drift error of MLE is less than 4 m in the vertical direction of walk trajectories, and the one of the proposed ADR/ZUPT is less than 3 m.
(e) In the 10 walking tests, the end position accuracy estimated by ADR/ZUPT is higher than MLE in eight groups and lower only in the sixth and the tenth tests. The estimated mean error of the end position by ADR/ZUPT is about 0.5 m less than the estimated one by the MLE. Figure 10 further demonstrates that the proposed approach is superior to MLE.

### 3.2. Closed-Loop Experiments

**Figure 11:**
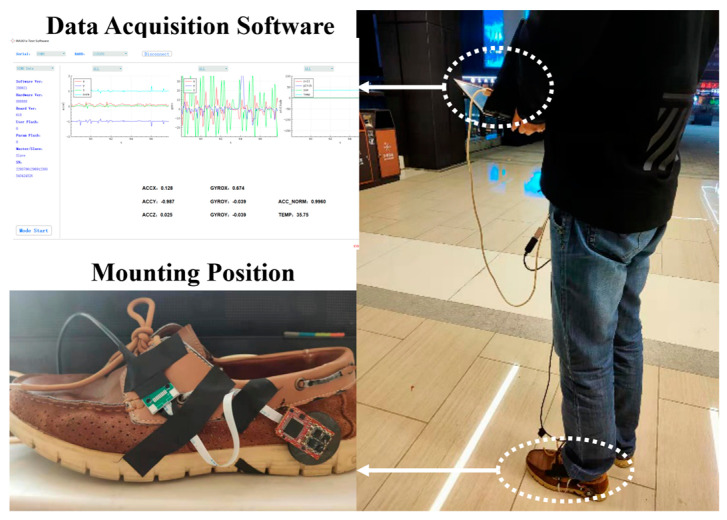
Closed-loop experiments scenes based on foot-mounted IMU array.

The total length of the designed closed-loop track is about 305 m and the walk tests are in a mall shown in Figure 11. Unlike the straight line experiments, the proposed method and MLE are compared and evaluated by the location deviation between the starting position and the end position due to lack of the reference trajectory.

**Figure 12:**
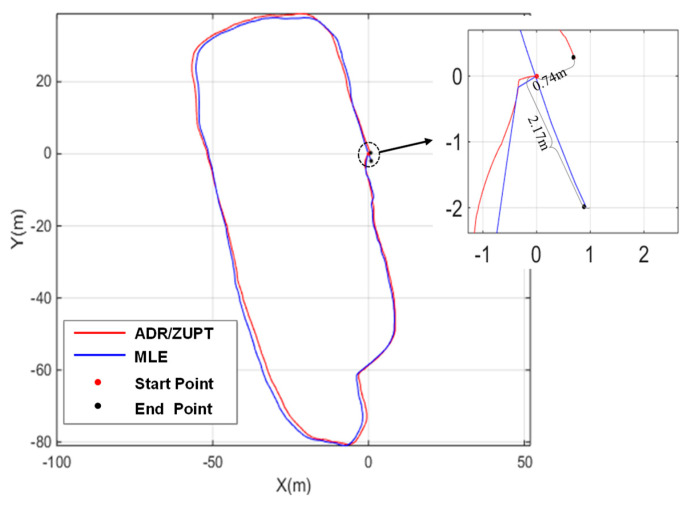
Positioning results of the IMU array by different methods in one closed-loop experiment.

The estimated result in one test is shown in Figure 12. In the test shown in Figure 12, the blue line represents the pedestrian track estimated by MLE, while the red one represents the pedestrian track estimated by ADR/ZUPT. The statistical results of the positioning errors in the closed-loop track estimated by two methods are shown in Figure 12, where the position closure error of each IMU in the designed array based on the traditional ZUPT is given.

**Figure 13:**
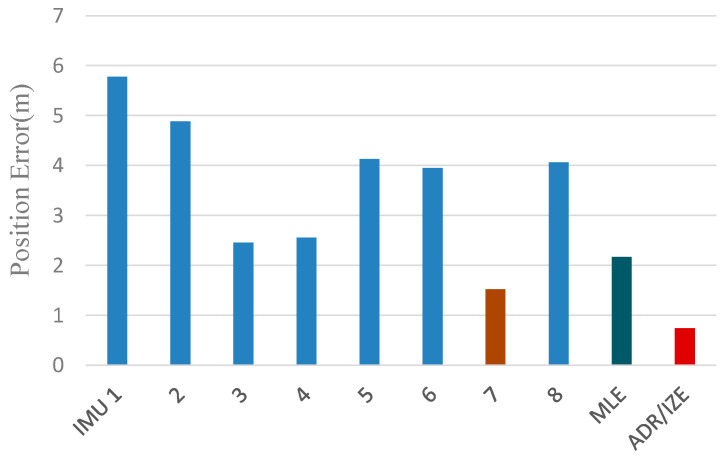
Positioning results of eight IMUs on the array in one closed-loop experiment.

Seen from the comparison in Figure 13, although the position closure error based on MLE is smaller than that of most IMUs in the array, it is larger than that of IMU 7. As is analyzed in the theoretical part mentioned above, the reason is that the position accuracy of the IMU array based on MLE is not higher than that of all the IMU, while the proposed ADR/ZUPT can adaptively eliminate the IMU with large errors in the fusion process, which wins it a higher accuracy. Meanwhile, since it adopts dead reckoning algorithm (ADR) based on LS, its position closure error is smaller than that of any IMU in the array.

Considering the difference between drift errors of IMU in different tests after powering on, 20 times of repeated powering on tests were carried out in the closed-loop track, including 10 times tests in line with the direction shown in Figure 6 and Figure 10 times in the opposite direction. In 20 statistical tests, the closed position accuracy was divided into several intervals shown in Table 2, and the test number in each interval was counted. The numbers in the table represent the number of tests.

It can be seen from Table 2 that, in the 20 tests, MLE fell in the range of <1% for 18 times, whose position accuracy was approximately 1% (1σ), while ADR/ZUPT fell in the range of <0.5% for 12 times and 19 times in the range of <1%, whose position accuracy could reach 0.6% (1σ) and 1% (3σ). Therefore, ADR/ZUPT is superior to MLE.

## 4. Conclusions

In order to balance the fusion weight of the IMU with different drift errors on the array and to improve the location accuracy of the pedestrian based on the foot-mounted IMU array, the paper proposes an ADR/ZUPT integrated scheme applied in the IMU array. On the one hand, the proposed ADR/ZUPT method optimizes the fusion scheme of the IMU array; on the other hand, it upgrades the traditional ZUPT model and improves pedestrian position estimation accuracy. In the scheme, the position estimator is extracted from the ZUPT model calculated independently by ADR. In ADR, firstly, IMUs with large drift errors are eliminated adaptively by the step size and the track angle calculated by each IMU. Then, the step size and the track angle are estimated by LS, and the position is calculated on the basis of dead-reckoning (DR) principle. Experimental results of the designed foot-mounted IMU array in two tracks verify that the accuracy of the proposed ADR/ZUPT is superior to that of the MLE. In the straight line experiments, the estimated mean error of the end position by ADR/ZUPT is about 0.5 m less than the estimated one by the MLE and has a smaller covariance distribution. In the closed-loop walking tracks, the positioning error of ADR/ZUPT is less than 0.6% (1σ), which is higher than 1% (1σ) of MLE.

## Figures and Tables

**Table 1:** Estimated error of end position in straight line experiments.

Method	Estimated Error of End Position (m)										
	Test 1	2	3	4	5	6	7	8	9	10	Mean
MLE	2.93	2.04	1.61	2.39	2.86	2.28	3.15	2.58	2.39	2.03	2.11
ADR/ZUPT	2.19	1.07	1.25	0.49	1.81	2.73	2.03	1.53	1.94	2.61	1.68

**Table 2:** Numbers of closed-loop experiments with different error percentage.

Percentage	IMU Label								Method	
	1	2	3	4	5	6	7	8	MLE	ADR/ZUPT
>3%	2	2	1	1	3	1	1	1	-	-
1–3%	15	17	11	8	16	18	8	19	5	1
0.6–1%	3	1	6	8	1	-	9	-	9	5
<0.6%	-	-	2	3	-	1	2	-	6	14
